# A Three-Year Retrospective Analysis: Do Nutritional and Immunological Indices Predict Postoperative Complications After Rectal Resection?

**DOI:** 10.7759/cureus.55700

**Published:** 2024-03-07

**Authors:** Rajeevan Philip Sridhar, Rajat Raghunath, Mark Ranjan Jesudason, Rohin Mittal

**Affiliations:** 1 General and Colorectal Surgery, Christian Medical College and Hospital, Vellore, IND

**Keywords:** morbidity, post-operative complications, immunological index, rectal resection, rectal cancer, nutritional index

## Abstract

Background

Nutritional and immunological indices, such as prognostic nutritional index (PNI), neutrophil-to-lymphocyte ratio (NLR), and platelet-to-lymphocyte ratio (PLR), have been used as predictors of outcomes and survival in a few cancers. However, the literature is unclear about their usefulness in predicting postoperative complications in rectal cancer resection operations. Additionally, the prescribed cut-off values as well as the timing of the tests for these indices vary among studies. We aimed to determine the role of PNI, NLR, and PLR in predicting postoperative complications in patients undergoing rectal resection.

Methods

This is a retrospective analysis from a colorectal unit of a tertiary care teaching hospital. All consecutive patients undergoing rectal resection for rectal cancer between April 2018 and March 2021 were included. PNI, NLR, and PLR were calculated from preoperative blood tests, and all morbidity and mortality within 30 days of operation were considered.

Results

A total of 202 patients were included. Three patients who did not have the necessary preoperative blood test reports were excluded. Of the remaining 199, 142 (71.4 %) were males. The mean age was 47.3 years. Of the patients, 13.6% (n = 27) had major morbidity (Clavien-Dindo grade 3-5), including one mortality. The mean PNI, NLR, and PLR were 49.9, 4.3, and 230.5, respectively. The mean PNI between the groups (no complication vs. complication) was 49.6 vs. 50.1 (p = 0.46) and the mean NLR between the same groups was 4.7 vs. 3.8, (p = 0.06), and both were not significant. The mean PLR between the groups (256.3 vs. 203.4, p = 0.01) was found to be significant but significance was not elicited when only major complications were considered. Hence, none of the indices were a good predictor of postoperative complications in our study.

Conclusion

The role of nutritional and immunological indices (PNI, NLR, and PLR) is limited in predicting postoperative morbidity in rectal resection operations.

## Introduction

Biomarkers such as nutritional and immunological indices have been studied for their role in predicting short and long-term outcomes after surgery, especially in cancer patients. Commonly studied indices include the prognostic nutritional index (PNI), neutrophil-to-lymphocyte ratio (NLR), and platelet-to-lymphocyte ratio (PLR), and these biomarkers use blood parameters, such as albumin reflecting the nutritional status and lymphocyte counts to reflect the immune status. Neutrophils and lymphocytes are significant in tumor immunology and inflammation [1]. These indices have also been studied to determine their role in projecting the tumor staging in cancer and response to various treatments, such as neoadjuvant systemic chemotherapy. A peripheral blood smear is easy to perform, and if these indices are found to be predictive of postoperative outcomes, it would allow for a quick, cost-efficient way to prognosticate rectal cancer operations. However, there is no clear consensus on the cut-off values among published studies and the timing of the tests to calculate the indices. We aimed to determine the role of preoperative PNI, NLR, and PLR in predicting postoperative complications in patients undergoing rectal resection for cancer.

This article was presented as a poster at the Digestive Disease Week 2023 on May 9, 2023, ACPGBI Annual Meeting 2023 on July 5, 2023, and at the home institution's annual research day [2].

## Materials and methods

This is a retrospective study from a colorectal unit of a tertiary care teaching hospital. All consecutive patients undergoing rectal resection for rectal cancer during three years between April 2018 and March 2021 were included. Inclusion criteria were all adult patients who underwent any rectal resection operation for rectal cancer in a single colorectal surgery unit during the study period. Patients who did not have the required blood tests performed in the preoperative period were excluded. Data were collected from electronic hospital records. Blood tests available within two weeks before the operation were used to calculate nutritional and immunological indices. All postoperative complications, including mortality up to 30 days following the operation, were recorded by patient chart review and classified based on the Clavien-Dindo classification of surgical complications [3].

PNI was calculated as serum albumin (g/L) + 5 × total lymphocyte count (109/L). NLR was measured by dividing the number of neutrophils by the number of lymphocytes: NLR = N/L [1]. The PLR was calculated based on peripheral platelet count (P; ×109/Liter) and lymphocyte count(L; ×109/Liter), using the formula: PLR = P/L [4].

The categorical variables in the study were reported in frequencies and percentages, and continuous variables were reported using mean ± standard deviation or median (range) as appropriate. The Pearson chi-square was used to find the association between the categorical variables. An Independent sample test was used to compare groups on the mean of the biomarker indices. A p-value less than 0.05 was considered statistically significant. The data entry was done using EpiData® 3.1 software and the statistical analysis was performed using SPSS® version 23.0 (IBM Corp., Armonk, NY).

## Results

A total of 202 rectal cancer patients underwent rectal resection operations in the study period and were eligible to be included in the study. Three patients who did not have the necessary preoperative blood test reports were excluded. Of the remaining 199, 142 (71.4 %) were males. The mean age of the study population was 47.3 years (SD: 13.9, range: 19 to 82). Other study patient characteristics are mentioned in Table 1.

**Table 1:** Study patient characteristics Values expressed as n (%).

Variables		N = 199
Age (years)	< or = 30	26 (13.1%)
	31 to 60	140 (70.4%)
	>60	33 (16.6%)
Gender	Male	142 (71.4%)
	Female	57 (28.6%)
Operation approach	Open	75 (37.7%)
	Laparoscopic	113 (55.8%)
	Laparoscopic converted open	13 (6.5%)
Operation type	Abdominoperineal resection	84 (42.2%)
	Anterior/low anterior resection	86 (43.2%)
	Anterior resection (Hartmann’s type)	23 (11.6%)
	Pelvic exenteration	2 (1%)
	Proctocolectomy	4 (2%)

A total of 100 patients (50.3%) did not have any complications and were grouped into the no complication group and the rest formed the complication group, which had 99 patients (49.7%). The grade of complications in the complication group is presented in Table 2. Twenty-two patients had surgical site infection, 18 had perineal wound infection, 11 had prolonged paralytic ileus, 11 had an intra-abdominal collection, nine patients had an anastomotic leak, eight had urinary tract infection, six had stoma-related complications, five had postoperative hemorrhage, four developed urinary retention, two had wound dehiscence, two patients developed upper respiratory tract infection, and one patient developed acute coronary event in the postoperative period. A total of 13.6% (n = 27) had major morbidity (Clavien-Dindo grade 3-5). One patient died of infectious complications.

**Table 2:** Table of complications Values expressed as n (%).

Grade of complication	Number of patients (percentage), N = 99
Clavien-Dindo grade 1	27 (13.6%)
Clavien-Dindo grade 2	45 (22.6%)
Clavien-Dindo grade 3	15 (7.5%)
Clavien-Dindo grade 4	11 (5.5%)
Clavien-Dindo grade 5	1 (0.1%)

The mean PNI, NLR, and PLR were 49.9 (SD: 5.4, range: 30.4 to 67.8), 4.3 (SD: 3.3, range: 0.9 to 29.7), and 230.5 (SD: 146.8, range: 11.8 to 1440.8), respectively. Using cut-off values of 47.1, 2.8, and 140.0 for PNI, NLR, and PLR, respectively, from available literature, chi-square p-values were 0.40, 0.43, and 0.84, respectively. None were found to be statistically significant in our study [5].

Though the mean PLR showed a statistically significant difference between the two groups, the statistical significance of mean PLR between the groups was not elicited when only major complications were considered.

**Figure 1:**
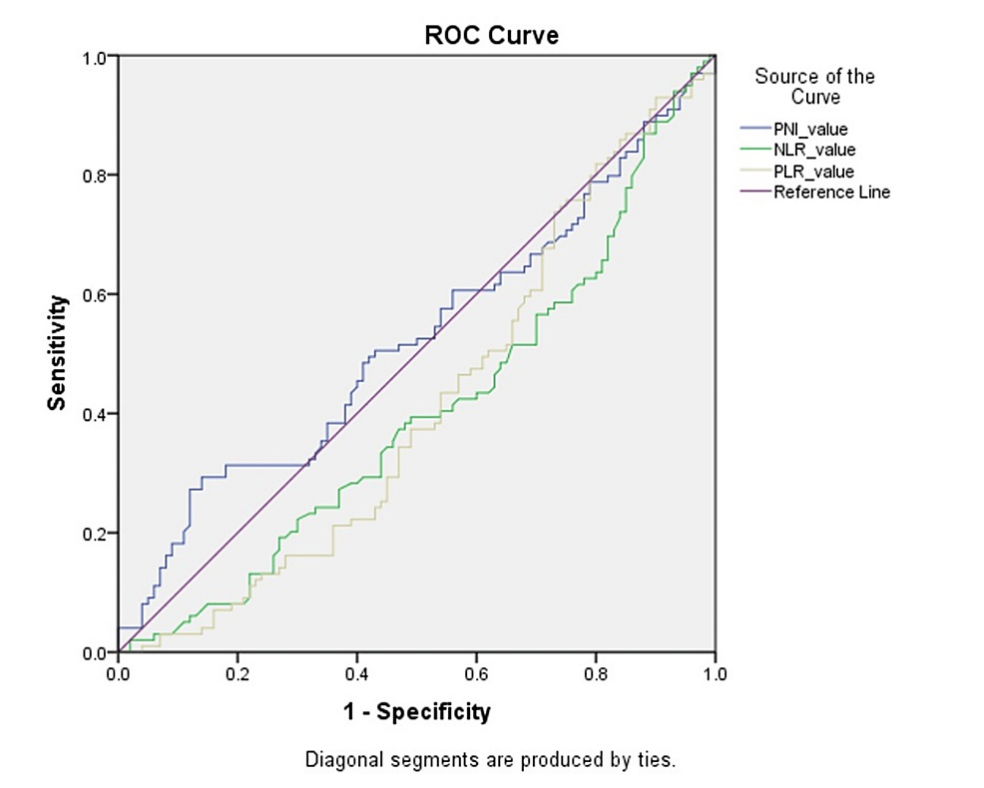
ROC curve for nutritional immunological indices ROC: receiver operating characteristic; PNI: prognostic nutritional index; NLR: neutrophil-to-lymphocyte ratio; PLR: platelet-to-lymphocyte ratio.

From Table 3 and Figure 1, we can infer that PNI, NLR, and PLR did not predict postoperative outcomes in our subset of patients who underwent rectal resection for rectal cancer.

**Table 3:** Comparison of means of indices between complication and no complication groups Values are expressed as mean ± standard deviation. Independent sample test used to calculate p-values. PNI: prognostic nutritional index; NLR: neutrophil-to-lymphocyte ratio; PLR: platelet-to-lymphocyte ratio.

Indices	Complication	No complication	P-value
Mean PNI	49.6 ± 4.6	50.1 ± 6.1	0.46
Mean NLR	4.7 ± 3.8	3.8 ± 2.7	0.056
Mean PLR	256.3 ± 178.9	203.4 ± 98.8	0.011

## Discussion

Novel nutritional and inflammatory biomarkers for prognosticating patients with cardiovascular disease as well as cancer have been described in the literature [4]. The PNI, NLR, and PLR are all derived from standard blood tests and are low-cost measurements that are already routinely used in clinical practice and can be calculated easily. They are useful in predicting survival outcomes in gastrointestinal cancers such as gastric cancer, pancreatic cancer, and colorectal cancer [6-9]. The role of these biomarkers in predicting morbidity has been studied in gastric and colorectal cancer and PNI was found to be useful [10,11]. However, there exists a wide variability in the described cut-off values among studies and the timing of the preoperative blood tests, especially in patients who receive neoadjuvant therapy.

Prognostic nutritional index (PNI)

PNI has been studied in gastric cancer and was found to be a useful predictor of long-term outcomes independent of the tumor stage. Patients with postoperative complications had a lower mean PNI compared to those without (p = 0.023) [12]. A low preoperative PNI was a predictor of postoperative complications in a retrospective study including colon and rectal cancer as a combined entity [13]. However, the mean PNI between the groups (no complication vs. complication) in our study among rectal cancer patients was 49.6 vs. 50.1 (p = 0.46). In our study, PNI was not predictive of postoperative complications in rectal cancer resections.

Neutrophil-to-lymphocyte ratio (NLR)

After a nonspecific systemic inflammatory response generated by a tumor, circulating neutrophil levels increase resulting in relative lymphocytopenia and an elevated NLR [14]. An association between a high preoperative NLR and a poor prognosis has been reported for several types of carcinomas, such as pancreatic, lung as well as colorectal cancer [15-17]. Li et al. conducted studies on a large group of patients suffering from colorectal cancer (n = 5336) after surgical resection in stage I-III and found that NLR with cut-off > 2.72 was an independent predictor of overall survival (OS) and disease-free survival (DFS) [9]. Although one retrospective cohort study on colorectal cancer has shown high preoperative NLR as a risk factor for major complications, no significant relationships were found between an elevated preoperative NLR and complication type [18]. The mean NLR between the two groups (no complication vs. complication) in our study was 4.7 vs. 3.8 (p = 0.056) and was not statistically significant.

Platelet-to-lymphocyte ratio (PLR)

One of the first cells to accumulate at the site of damage are platelets and they release their granule contents locally initiating an inflammatory cascade. Together with other immunologically competent cells, they form the “tumor microenvironment” [19]. Increased PLR in patients with colorectal cancer was found to be associated with poor prognosis and an increased risk of distant metastasis [8]. A low PLR was identified to be an independent risk factor for predicting postoperative complications after curative gastrectomy for cancer in the literature [20]. The mean PLR between the two groups (no complication vs. complication) in our study was (256.3 vs. 203.4, p = 0.011) and was found to be statistically significant; however, the significance was not elicited when only major complications were considered.

Hence, none of the indices were a good predictor of postoperative complications in our study on rectal cancer operations. The varying findings on the role of nutritional immunological indicators in predicting outcomes are due to varied cut-offs used in different studies and no consensus on the timing of the blood tests used to calculate the indices. Another factor that may contribute to this is the effect of neoadjuvant chemotherapy and radiotherapy on the nutritional and immunological blood test parameters used in preoperative assessment. These can be overcome by standardizing the protocol for the time of testing and performing a prospective trial powered to allow for a subset analysis of rectal cancer patients who did and did not receive preoperative chemotherapy and radiation. Longer follow-up would be recommended to predict the survival analysis and recurrence outcomes.

Limitations

The main limitation of the study was its retrospective nature of data collection. Some patients were excluded since blood tests were not available in the specified preoperative period. The blood tests done in the preoperative period were done on varying days within the two weeks. Some patients had neoadjuvant therapy, which can cause lower white blood cell and platelet counts. Also, there was variation in the waiting time after neoadjuvant chemotherapy and radiotherapy to the operation, which may have influenced the immunological biomarker tests. These may also contribute to a bias in the interpretation of the results.

## Conclusions

In our study, the nutritional immunological indices did not predict postoperative complications in rectal cancer resection operations. The role of nutritional and immunological indices (PNI, NLR, and PLR) is limited in predicting postoperative morbidity in rectal resection operations.
